# The role of appendectomy and cholecystectomy in the pathogenesis of colorectal carcinomas

**DOI:** 10.1016/j.amsu.2021.102991

**Published:** 2021-11-03

**Authors:** Miklós Mándi, György Keleti, Miklós Juhász

**Affiliations:** General, Vascular and Thoracic Surgery Unit, Bajcsy-Zsilinszky Hospital, Budapest, Hungary 89-91, Maglódi Street, H-1106, Hungary

**Keywords:** CRC, Colorectal carcinoma, LC, Laparoscopic cholecystectomy, BMI, Body mass index, EESzT, National Electronic Health Service Space, RCC, Right-sided colon cancer, LCC, Left-sided colon cancer, Appendectomy, Cholecystectomy, CRC (Colorectal carcinoma), Carcinogenesis

## Abstract

**Background:**

Several alterations in the gastrointestinal tract which occur after appendectomy or cholecystectomy have been suggested to raise the risk of developing colorectal carcinoma. Given the frequency that these procedures are performed, we sought to determine whether a history of either cholecystectomy or appendectomy increased the risk of future colorectal carcinoma.

**Methods:**

We determined the number of patients with a history of appendectomy and cholecystectomy who developed colorectal carcinoma between January 2018 and February 2021, as well as the latency time between the two diseases. Secondly, we carried out a data-collection spanning 15 years after the primary surgery (January 2005–December 2006).

**Results:**

The post-cholecystectomy state is significantly more frequently observed in patients treated for colorectal carcinomas (both male and female), especially among those who developed right-sided or left-sided colon cancer, as opposed to anorectal cancer (*p* = 0.53). However, the time elapsed between the two diseases is 20–25 years, which appears to be markedly long regarding such a multifactorial disease as the colorectal carcinoma. No similar extra risk was observed among patients having appendectomy. Secondly, we found no extra risk during the first 15 years after cholecystectomy.

**Conclusion:**

Although a statistically higher risk of colon cancer is observed after the removal of the gallbladder, but the latency time is long. Thus, cholecystectomy may not be an independent risk factor for colorectal carcinogenesis. Altogether, the patient is not exposed to a higher risk of colorectal carcinogenesis after having cholecystectomy.

## Highlights

Retrospective study

• Cholecystectomy is a risk factor for CRC in male and female, and for tumors of the right and the left side of the colon.
• Appendectomy was not significantly associated with an increased risk of developing CRC.
• The time between CRC and cholecystectomy is significantly shorter than that after appendectomy, yet more than 20 years.

Retrospective cross sectional study

• The risk for CRC was not significantly elevated compared to varicectomy, hernia repairs or appendectomy over 15 years.

## 1 Introduction

The etiology of colorectal carcinoma has been extensively studied. Divers post-cholecystectomy changes have been suggested to raise the risk of the development of colorectal carcinomas (CRC). For example, the altered enterohepatic circulation of bile acids after cholecystectomy might lead to a dysbacteriosis [1] and the modified gut flora may produce prominent carcinogenic agents [2,3] from the constantly secreted bile acids, posing a substantial risk to develop CRC. Other authors who studied the role of immunologic factors in the development of CRC have suggested that the impaired intestinal immune status after removing the vermiform appendix (‘abdominal tonsil’) can lead to a higher risk for CRC [4]. Previous studies have yielded controversial results on the relationship between appendectomy or cholecystectomy and CRC: a number of reports confirmed the possibility of a higher risk [5,6], but others have concluded that there was no significant additional risk at all [7,8]. The metaanalysis of a great variety of studies showed notable geographic differences for this effect. The post-cholecystectomy risk of developing CRC is observed predominantly in the Western countries [6], while in the South-Eastern Asian population the results are inconsistent. A set of studies in Korea revealed that the risk of CRC increased 1–5 years after cholecystectomy, while no difference was detected in the long run [9,10]. A recent study reported that the risk of CRC was higher after the removal of the gallbladder [11]. A novel report from Szeged, Hungary noted a pronounced left to right shift in the localization of colorectal tumors that developed after appendectomy or cholecystectomy [12].

The incidence of CRC in Hungary reached dangerously high levels in the 2010's [13,14]. According to the Hungarian National Cancer Registry's data, the number of new CRC cases exceeded 10,000 per year: 5768 men and 4786 women were diagnosed with CRC in 2018 [13]. Likewise, Hungary ranked first on the World Cancer Research Fund's worldwide Colorectal Cancer statistics chart in 2018 with 51.2 cases/100.000 people [men (1st position): 70.6/100.000 and women (2nd after Norway): 36.8/100.000] [15]. In the same year, about 6000 patients died of colorectal cancer representing an extremely high death rate (nearly 60%).

In Hungary, one of the most common general surgical treatments is cholecystectomy. 23–24,000 such operations were performed annually between 2018 and 2020 [16], including 20–21,000 laparoscopic cholecystectomies (LC), and approximately 10–15% of the entire population is affected with the gallstone disease. On the other hand, the most common emergency surgery is appendectomy, approximately 16% of the population undergoes appendectomy during their lifetime. There are 9–10,000 appendectomies per year in Hungary [16] and the proportion of the laparoscopic approach has been increasing steadily, reaching 5–6000 annually [16] in 2018–2020.

Since the laparoscopic approach to cholecystectomy has become so widespread and is available nationwide, the removal of the gallbladder became an easily feasible procedure, thus the indication criteria for cholecystectomy have been broadened. In the daily practice, the verification with ultrasound of sludge or gallstones as well as other benign diseases of the gallbladder including cholesterol polyposis is sufficient to propose the operative treatment based on the guidelines provided by the major textbooks [17,18] and local protocols (example: [19]). Thus, in most cases, the indication for cholecystectomy is facultative and the procedure is carried out on healthy patients without any symptoms as a means of prevention of a possible bilious attack, cholecystitis, cholecystopancreatitis, choledocholithiasis or worst of all, gallbladder cancer.

Likewise, the spreading of the laparoscopic technique among emergency operations has led to thorough changes in the strategy of removing the vermiform appendix: decades ago, in the era of open surgery, the negative appendectomy ratio was a measure of the diagnostic accuracy of a surgeon or a whole surgery unit, thus the indication for appendectomy had to be well considered. At present, however, we tend to remove even the macroscopically negative, non-inflamed vermiform appendix in case of a diagnostic laparoscopy for lower quadrant pain.

The goal of this study is to determine whether the aforementioned large number of surgeries carried out, often for facultative indications, might expose a patient to a higher long-term risk of developing CRCs.

## 2 Methods

### 2.1 The retrospective study

**Fig. 1:**
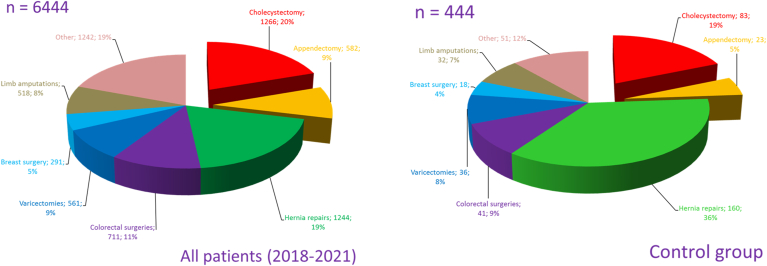
The distribution of the different types of surgical treatments (n = 6444) practised on the Surgery Unit of the Bajcsy-Zsilinszky Hospital (between January 1, 2018 and February 15, 2021) and the components of the control group (n = 444).

We searched our surgical database at the Bajcsy-Zsilinszky Hospital (Budapest, Hungary) for all patients who underwent surgical treatment between January 1, 2018 and February 15, 2021. During this period, 458 surgeries were carried out for tumors of the colon or the anorectum (CRC group). 259 of the treated patients were male and 199 female. In approximately one third of cases, only the type of the previous surgery was known without an exact date, therefore the data concerning these cases were procured by reasonable estimation. When determining the estimated time, the patient's age was taken in consideration along with the typical age of having an appendectomy (age 5–25) or cholecystectomy (age 20–40). During the study period, a total of 6444 surgeries were carried out within our surgical unit, of which 444 comprised the control group. Control subjects were randomly chosen, yet the final group was matched to the study subjects by age and gender (see Table 1). Smoking, alcohol abuse and CRC present in the patient's family were significantly more common in the CRC group. Obesity showed a controversial distribution between the control and CRC group. Significantly higher BMI is associated with CRC in the entire group and the men's group, however it showed a reciprocal relation in women. In addition, the composition of the control group mirrors the wide range of surgical treatments practiced on our Surgery Unit (see Fig. 1). The study was approved by the IRB of the Bajcsy-Zsilinszky Hospital. Each and every data was obtained anonymously, and all patients have signed their consent to have their data used for statistical analysis upon admission to our Unit.

**Table 1:** The different parameters of the CRC and control groups formed from patients treated on the Surgery Unit of the Bajcsy-Zsilinszky Hospital between January 1, 2018 and February 15, 2021: number of patients, average age, M/F ratio, BMI, rate of smoking, alcohol abuse and CRC in the family, the proportion of appendectomy or cholecystectomy in the anamnesis and the latency times.

	Appendectomy (%)		Cholecystectomy (%)	
	Control	CRC	Control	CRC
**All patients**	15.32	14.19	9.91	17.03
**Male**	11.67	11.2	3.89	10.04
**Female**	20.32	18.09	18.18	23.9

### 2.2 The retrospective cross sectional study

The retrospective cross sectional study included all patients treated with cholecystectomy or appendectomy on our Surgery Unit between January 1, 2005 and December 31, 2006 who were followed until April 2021 compared to two separate control groups. The first was composed of patients who underwent hernia repair and the second of patients who underwent varicectomy. These two groups were chosen because neither operation affects the intestinal tract. Patients who had intestinal resection during the operation were excluded from the ‘hernia repair’ group. The data was collected from various sources, primarily from the hospital's own digital data base, and also from the nationwide accessible National Electronic Health Service Space (EESzT) that was launched in July 2017 and that contains extensive healthcare data deriving from all healthcare services in Hungary (public and private equally). Table 2 shows the proportion of evaluated cases, which was lowest in the appendectomized group: 47 patients out of 232 were lost to follow after the appendectomy, resulting in a group of 185 (79.7% of the whole appendectomized group). The rate of successful evaluation varied between 84 and 90.5% in the other three groups. Criteria for a successful evaluation of a case were the following: validated appearance in the hospital's own digital data-base as recently as the 1st quarter of 2021, or a proof of the death of the patient in our data-base or on the EESzT, or any recent data concerning the patient found on the EESzT.

**Table 2:** The parameters of the different groups formed from patients treated on our Surgery Unit in 2005–2006.

	Number of evaluated patients	Proportion of evaluated patients (%)	Average age (years)	M/F ratio	Proportion of deaths (%)	Proportion of cholecystectomy in patient's history (%)	Time elapsed since cholecystectomy (years)	Proportion of appendectomy in patient's history (%)	Time elapsed since cholecystectomy (years)
**After varicectomy (control group) n = 176**	159	90.34%	50	44/115	10.06%	6.81%	9.56	15.91%	31.1
**After hernia repair (control group) n = 179**	155	86.59%	53,2	112/43	25.16%	7.74%	10.56	14.19%	30.31
**After appendectomy n = 232**	185	79.74%	38,535	84/101	7.57%	5.17%	13.42	X	X
**After cholecystectomy n = 427**	361	84.54%	51.57	81/280	12.74%	X	X	17.17%	33.25

### 2.3 Statistics

The comparison of the data obtained in the different groups was made by using the *Medcalc.org*'s online calculators. When the results were expressed in percentage, the calculator that compares proportions was used:

https://www.medcalc.org/calc/comparison_of_proportions.php.

In studies where elapsed time was compared, the

https://www.medcalc.org/calc/comparison_of_means.php calculator was chosen to compare the different groups' mean time values and generally 10% of the means’ value was applied as standard deviation. For “N-1” Chi-squared test and for comparing means, the computational notes are given on the website. When generating the *p*-value, the null hypothesis is that the difference of the obtained values of the two groups is 0. A *p*-value that is less than 0.05 (*p* < 0.05) was considered statistically significant.

Approval was obtained from the Institutional Review Board (IRB). This case series has been reported in line with the STROCSS 2019 Guideline [20] and was registered in accordance with the declaration of Helsinki (ID: researchregistry7126).

Https://www.researchregistry.com/register-now#home/registrationdetails/6135d1b00004bc001f36f8e0/

## 3 Results

### 3.1 The retrospective study

**Fig. 2:**
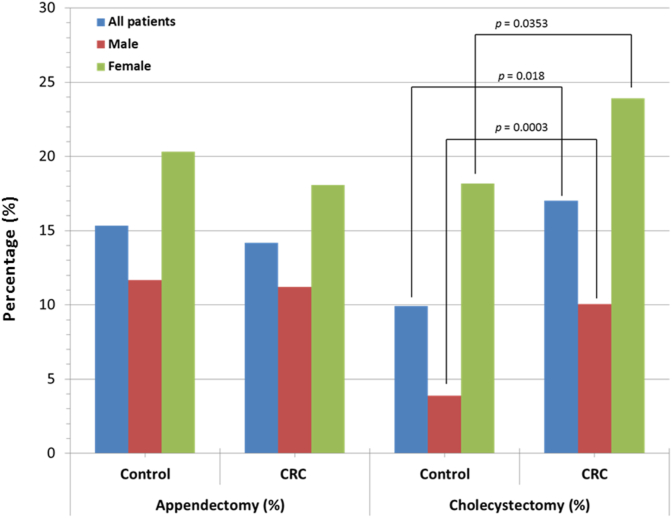
The proportion of appendectomy or cholecystectomy in the history of male and female patients. Significant differences are marked by indicating the *p* values.

The results gathered in our retrospective study are summarized in Table 1. 14.2% of all patients in the CRC group had a previous appendectomy; the rate in men was 11.2%, as opposed to 18.1% among women. The values are similar in the control group (15.3% overall, 11.7% in men and 20.3% in women). As shown on Fig. 2, there was no statistically significant difference between the two groups. The elapsed time between the two diseases is extremely long, 40.9 years for the entire CRC group and 47.3 years in the control group (see Table 1).

**Fig. 3:**
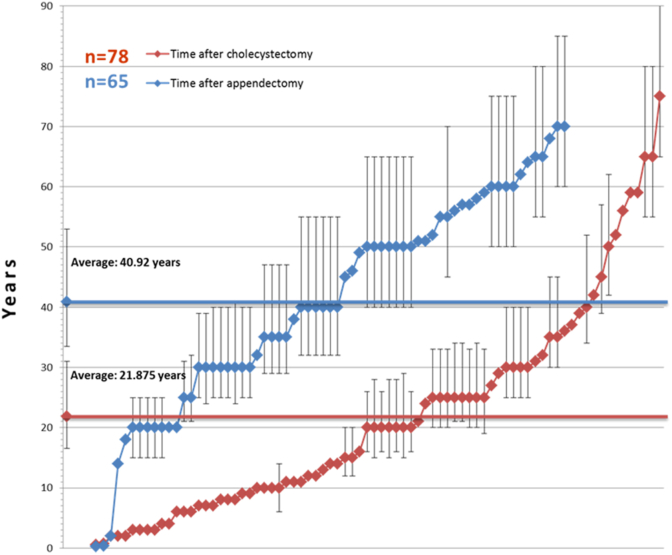
The latency time between appendectomy or cholecystectomy and the diagnosis of CRC in the individual cases. The average latency time after appendectomy is about 17 years longer than after cholecystectomy (*p* < 0.0001).

Our data indicate a different behavior of the post-cholecystectomy state (Table 1). The latency time between cholecystectomy and the diagnosis of CRC is substantially shorter, 21.9 years in the entire CRC group and 22.6 years in the control group (Fig. 2). The likelihood of having a cholecystectomy is 17% in the CRC group versus 9.9% in the control group (*p* = 0.0018). Among male patients, the corresponding values are 10% vs. 3.9% (p = 0.0062) and in women, 23.9% vs. 18.2% (*p* = 0.0353). The latency times to diagnosis of CRC are shown in Fig. 3. Error bars were added where the date of the first operation could not be specified and the data-point is a result of reasonable estimation. Appendectomy and cholecystectomy had previously been performed in 65 and 78 cases in the CRC group. Both procedures were performed in 10 patients and in 3 cases, the appendectomy was performed simultaneously with the colorectal resection or the CRC was identified during the appendectomy and radical resection was made within 2 years. The latency time after the appendectomy is less than 20 years in 2 patients (14 and 18 years) and more than 20 years in the remaining 60 cases with an overall average latency time of 40.9 years. On the other hand, 5 patients had colorectal surgery less than 2 years after cholecystectomy and in 32 cases, the latency time varies between 3 and 19 years. The remaining 41 patients had their cholecystectomy done more than 20 years before the diagnosis of CRC making the average latency time 21.9 years. The aforementioned two averages differ significantly (*p* < 0.0001). As seen in Fig. 3, the number of short latencies after appendectomy and cholecystectomy markedly differ from each other in accordance with previous reports [10], where the authors concluded, that a higher risk for CRC is observed 1–5 years after cholecystectomy.

**Fig. 4:**
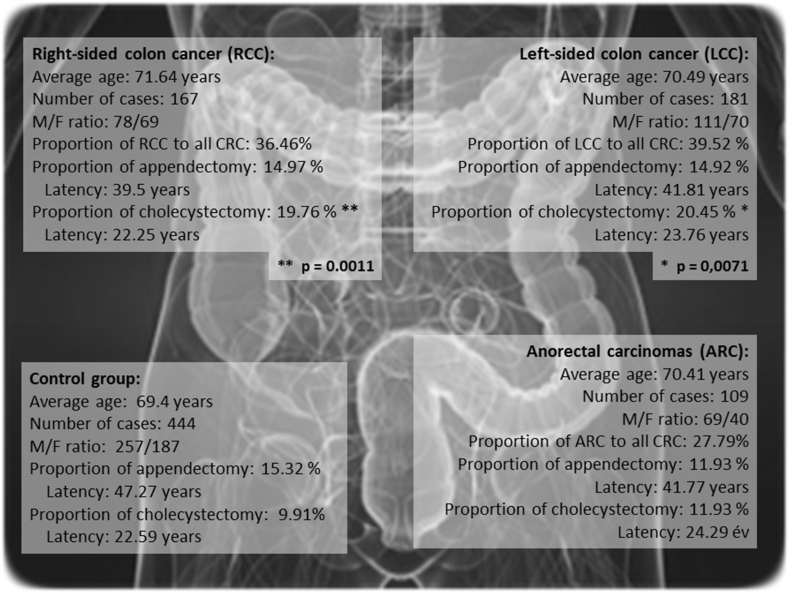
The latency times and the distribution of appendectomy or cholecystectomy in the anamnesis depending on the different localizations of CRC. Cholecystectomy figures significantly more frequently in the anamnesis of patients having right-sided and left-sided colon cancers (but not anorectal tumors), the *p* values are 0.0011 and 0.0071, respectively.

The locations of the CRC are shown in Fig. 4. Right sided colic cancer (RCC) was found in 167 cases, left sided cancers (LCC) were found in 181 patients and anorectal carcinoma (ARC) was found in 109 cases. The rate of appendectomy is higher as one moves from the anorectum towards the right colon and parallel to this, the latency time diminishes slightly. The aforementioned value is 15.3% in the entire CRC group and 11.9% in those diagnosed with ARC, 14.9% in LCC and 15% in RCC. On the other hand, CRC is preceded by cholecystectomy in 9.9% of the cases in the control group and 11.9% among patients with ARC. Moving towards the left and further to the right colic areas, a substantial rise is seen (LCC: 20.5%, RCC: 19.8%) that is statistically significant (*p* = 0.0071 for LCC and *p* = 0.0011 for RCC). Thus, cholecystectomy is significantly more frequent in tumors located on either the right side, or the left side of the colon. Our findings are in accordance with the results published in 2020 by the Surgery Clinic in Szeged [12] and other publications as well [21].

### 3.2 The retrospective cross sectional study

**Fig. 5:**
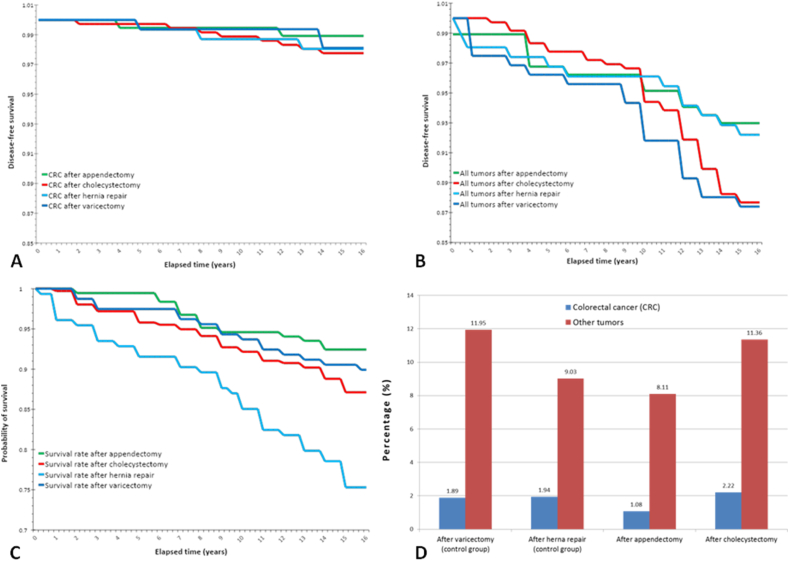
The rate of CRC-free survival (**A**), tumor-free survival (**B**) and overall survival (**C**) after the primary surgery, as well as the percentage of diagnosed CRCs and all other types of tumors (**D**) during the first 15 years after the primary operation in 2005–2006.

To further evaluate our findings, we designed a retrospective cross sectional study to gather more evidence regarding a possible carcinogenic role of cholecystectomy (Table 2). 8 out of the 361 patients who underwent cholecystectomy have developed colorectal carcinoma during the observation period (2005–2020) with an average elapsed time of 9.4 years, while other types of tumors (except localized skin tumors) were diagnosed in 41 cases (10 years on average after the surgery). Among the 185 patients who had an appendectomy, CRC was diagnosed in 2 cases. On the other hand, CRC as a synchronous tumor was found during the operation for right hypogastric pain syndrome or appendectomy was made at the time of a colorectal resection in 4 cases, moreover 1 patient had a rectal resection 7 years before the appendectomy. 15 patients have developed various other types of malignancies after appendectomy. In the post-varicectomy group (n = 158), 3 cases of CRCs and 19 cases of other types of tumor have been observed, average elapsed time after the surgery was 11 and 8.4 years respectively. 3 patients out of 155 in the hernia repair group have been diagnosed with CRC after the operation (on average 6.6 years later) and 1 of them had cholecystectomy prior to the hernia repair. 14 other types of tumors appeared in the hernia repair group. In Fig. 5, we plotted the CRC-free survival **(A),** the malignant tumor-free survival **(B)** and the overall survival **(C)** rates using the Kaplan-Meier method. The aforementioned synchronous tumors were omitted from the figures since these tumors developed before the surgical treatments took place, thus no postoperative changes contributed to their pathogenesis. Moreover, the results found in the different groups were compared (Fig. 5 D). The rate of the development of CRC within 15 years after the first surgery is almost identical, 1.88% and 1.93% in the two control groups (varicectomy and hernia repair respectively), while it is somewhat lower (1.1%) after appendectomy and moderately higher in the post-cholecystectomy group (2.2%). There were no statistically significant differences between the different groups, thus there was no surge in the development of CRC during the first 15 years after cholecystectomy. Also, the lower value seen in the post-appendectomy group does not reflect a significant difference. Likewise, the rate of the other types of tumors after the primary operation shows similar tendencies: 11.95% after varicectomy and 9% after hernia repairs, somewhat less, 8.1% after appendectomy and 11.4% after cholecystectomy.

## 4 Discussion

In the present study, we investigated the potential role of a cholecystectomy or an appendectomy as a risk factor for colorectal carcinogenesis. The retrospective approach delivered proof of cholecystectomy being a risk factor among both male and female patients, as well as for tumors of both the right and the left side of the colon, but not for the anorectum. However, the rate of being appendectomized was not significantly associated with an increased risk of developing CRC. The elapsed time until the appearance of a CRC after cholecystectomy is significantly shorter than that after appendectomy, yet it is still well over 20 years, which is a rather long period, thus making it questionable that cholecystectomy should be considered as an independent risk factor in a multifactorial disease such as CRC. According to WHO's Hungarian Country Health Profile concerning year 2017, most of the non-hereditary risk factors of CRC are present profoundly and prominently in the Hungarian society: 26% of the adults are smokers, 21% are overweight and 38% are heavy drinker or alcoholic [22]. Moreover, the so called Hungarian diet, Hungarian cuisine that is dominated by red meat (beef), pork and is rich in fat, grease and hot spices makes matters worse. Stressful and sedentary lifestyle adds up even more risk for CRC. Thus, the contribution of cholecystectomy to the development of CRC is subdued by the aforementioned factors.

We also investigated the possible carcinogenic role of appendectomy and/or cholecystectomy by means of a retrospective longitudinal study. The nearly 15-years period after the primary surgery did not prove to be long enough to reveal the assumed increased carcinogenic effects of cholecystectomy. The latter assumption seemed plausible based on Fig. 3, where CRC diagnosed less than 15 years after cholecystectomy was found in just a small number of cases and by far more often, than in the case of appendectomy. Unfortunately, we were not able to choose a longer period of time for our retrospective longitudinal survey since the patients’ data is only available electronically (on-line) from 2005 and the accuracy of off-line data acquisition is much too doubtful.

Key limitations of the current study include the relatively short (15 years long) follow-up time of the retrospective longitudinal study that could not approach the mean latency between cholecystectomy and CRC (21.6 years). This period cannot be extended due to the lack of the digitalization of the older data. Another weak point is the high proportion of estimated values when determining the date of any appendectomy and/or cholecystectomy in the patient's history. On the other hand, our study takes into consideration the importance of the latency time for the first time and provides evidence on the role of cholecystectomy and appendectomy from two different angles. Another key limitation to this study may be its single centered nature, although our hospital covers 3 different districts within the capital city as well as suburban territories, sleeping cities outside Budapest along with rural areas and small villages. Thus, patients treated in our hospital represent all classes and all segments of the Hungarian society which enables us to extrapolate our data. In summary, our conclusion is that although a statistically significant relationship exists between the post-cholecystectomy state and the development of CRC, this effect may not prevail directly due to the extreme long latency time. Nonetheless, when surgeons indicate cholecystectomy in cases with normal gallbladder, they should be aware of its possible adverse long-term effects.

## Funding

None.

## Ethical approval

IRB committee of the Bajcsy-Zsilinszky Hospital 19-05/2021.

## Author contribution

Miklós Mándi M.D., Ph.D. study concept, data collection, data analysis and interpretation, writing the paper. Prof. György Keleti M.D. data interpretation, writing the paper, study concept, Miklós Juhász M.D. data interpretation, study concept.

## Registration of research studies

1. Name of the registry: The role of appendectomy and cholecystectomy in the pathogenesis of colorectal carcinomas
2. Unique Identifying number or registration ID: researchregistry7126
3. Hyperlink to your specific registration (must be publicly accessible and will be checked): https://www.researchregistry.com/browse-the-registry#home/registrationdetails/6135d1b00004bc001f36f8e0/

## Guarantor

Dr. Miklós Mándi.

## Declaration of competing interest

None.
